# Editorial: Lipids in Cyanobacteria, Algae, and Plants—From Biology to Biotechnology

**DOI:** 10.3389/fpls.2021.834384

**Published:** 2022-01-11

**Authors:** Juliette Jouhet, Mie Shimojima, Koichiro Awai, Eric Maréchal

**Affiliations:** ^1^Laboratoire de Physiologie Cellulaire et Végétale, CNRS, CEA, INRAE, Université Grenoble Alpes, IRIG, CEA Grenoble, Grenoble, France; ^2^School of Life Science and Technology, Tokyo Institute of Technology, Tokyo, Japan; ^3^Department of Biological Science, Faculty of Science, Shizuoka University, Shizuoka, Japan

**Keywords:** alga lipid, plant lipid, fatty acid, glycerolipid, sphingolipid, sterol, triacylglycerol

Lipids are small amphipathic molecules, harboring a hydrophobic moiety and more or less developed hydrophilic regions, with a broad diversity of chemical structures, ranging from fatty acids and acyl-lipids, like glycerolipids, sphingolipids, wax esters, etc, to isoprene-derived structures that include polycyclic forms like sterols. Lipids are therefore generated by a variety of distinct pathways within carbon metabolism but they converge to populate (and generate) specific cell structures, most notably cell membranes, lipid droplets and extracellular barriers. The composition of each of these compartments is not random, but finely controlled, regulated and tuned, reflecting important biological roles for each lipid class and the existence of mechanisms regulating the corresponding metabolic routes in a coherent manner. In addition to this contribution to cell architecture, and to a multitude of functions including cell signaling, lipids are energetically dense and store carbon and energy in the form of triacylglycerol, or oil. Photosynthetic cells contain the common lipids conserved from bacteria to eukaryotes including fungi and animals, as well as unique classes of lipids, which are absent or under-represented in non-photosynthetic systems. These lipids play specific roles in photosynthetic organism. Cyanobacteria, algae and plants have lifestyles marked by a strong exposure to environmental variations, which they need to acclimate and adapt to, strikingly at the level of their lipid composition. Eventually, photosynthetic organisms are established sources of lipids for humans, for food, health, green chemistry and energy sectors.

The multidisciplinary study of plant lipids in general sense, from basic to applied sciences, is achieved by a scientific community, who gather every 2 years at the International Symposium on Plant Lipids (ISPL). ISPL meetings were started in 1974 by an initiating Plant Acyl Lipid Symposium in Norwich, organized by Terry Galliard. In 1976, the Karlsruhe Symposium extended its scope to all other plant lipids, such as isoprenoid lipids (sterols, carotenoids and prenyl side chains of chlorophylls and prenylquinones) and lipid polymers. The 24th ISPL was organized in July 2020 as a web-based event in the context of the Covid-19 pandemic. The present Research Topic compiles key contributions in the scope of this meeting, addressing lipids in cyanobacteria, algae, and plants, from biology to biotechnology.

At the front of basic knowledge, whereas it is commonly considered that the components of some pathways have been elucidated in plants and algae, others remain puzzling. This is particularly the case in three areas. Firstly, sterols are marked by the existence of complex and sometimes diverging metabolic pathways in different photosynthetic lineages. Darnet et al. provide a comprehensive review on phytosterol in the evolution of photosynthetic eukaryotes, and Girard et al. address more particularly a possible cycloartenol-to-cholesterol route in brown algae. Secondly, our view of the biosynthesis and role of sphingolipids is incomplete. In this regard, the development of fumonisin B1 to disrupt sphingolipid biosynthesis can be considered as an interesting tool for functional studies in plants, as overviewed by Zeng et al. Thirdly, the determination of the composition of the cytosolic compartment loaded with triacylglycerol, i.e., the lipid droplet (LD), and the deciphering of its biogenesis, need also important efforts. Indeed the lack of conservation of LD associated proteins in the course of evolution limits knowledge transfers from non-plant models. Filling knowledge gap on LD is also critical for biotechnological and agronomical purposes detailed below. Doner et al. describe how EARLY RESPONSIVE TO DEHYDRATION 7 localizes to LD in *Arabidopsis* and Guéguen et al. review our current knowledge on LD architecture and dynamics in Stramenopiles, a group of complex algae.

In both plant and algal models, lipid remodeling proves to be one of the very first responses to abiotic stresses. Lipid metabolism and cell compartmentation are therefore critical for the acclimation and adaptation of photosynthetic organism to environmental changes. Liu et al. show how galacto- and phosphoglycerolipids respond to salt stress in the crop *Glycine max*. Degraeve-Guilbault et al. decipher the processes involved in the control of acyl desaturation level in the green alga *Ostreococcus tauri* in response to temperature variations. Kokabi et al. detail acyl-lipid remodeling in *Lobosphaera incisa* in response to nitrogen and phosphorus availability, whereas Devadasu and Subramanyam highlight an enhanced accumulation of triacylglycerol in *Chlamydomonas reinhardtii* upon iron deficiency. This later work illustrates how a natural response of an alga to a nutrient stress may be translated in an industrial process to enhance algal oil production. Likewise, triacylglycerol and astaxanthin accumulation in *Haematococcus pluvialis* upon acetate stress, detailed by Hu et al. could be useful for biotechnological applications.

Based on robust knowledge on fatty acid and acyl-lipid metabolism, it is possible to develop strategies to improve the quality and quantity of triacylglycerol (oil) in oleaginous algae and crops. Concerning acyl-lipid metabolism in the general scheme of carbon partitioning, Zhai, Keereetaweep, Liu, Xu, et al. analyze the impact of sugar signaling on fatty acid biosynthesis in plants, whereas Sato and Toyoshima provide a remarkable analysis of carbon flow in starch and lipids in *Chlamydomonas debaryana* based on isotopic labeling experiments. Azlan et al. analyze the expression of acyl-CoA binding proteins in soybean to provide useful information for future biotechnological improvements. Behera et al. evaluate the role of WRINKLED2 in triacylglycerol production in avocado, providing new targets to enhance oil biosynthesis. Based on the stabilization of WRINKLED1 by trehalose-6-phosphate, Zhai, Keereetaweep, Liu, Feil, et al. show that the heterologous expression of bacterial trehalose-6-phosphate synthase increases oil accumulation in seeds and vegetative tissues. Eventually, Jarvis et al. explore Crispr/CAS9-based edition of specific genes to increase oleic acid content in pennycress seed oil.

Altogether, the contributions to this Research Topic illustrate the dynamic international efforts to fill gaps in knowledge on lipid biology in algae and plants, as well as attempts to translate this knowledge into biotechnological applications. The role of lipids in the early response of photosynthetic organisms to abiotic stresses also highlight how lipids are critical in the context of climate change. There is no doubt this question will become central in the near future.

## Author Contributions

All authors have contributed to the writing of the editorial.

## Funding

JJ and EM are supported by the National Research Agency (BLinK ANR-18-CE92-0015, Alpalga ANR-20-CE02-0020, GlycoAlps ANR-15-IDEX-02, GRAL Labex ANR-10-LABEX-04, and EUR CBS ANR-17-EURE-0003) and Institut Carnot 3BCAR. EM was supported by the Kilian Jornet Foundation. MS and KA are supported by the Japan Society for the Promotion of Science (JSPS) KAKENHI (JP18H03941). MS was supported by JSPS KAKENHI (21K05325). KA was supported by JSPS KAKENHI (20K06683).

## Conflict of Interest

The authors declare that the research was conducted in the absence of any commercial or financial relationships that could be construed as a potential conflict of interest.

## Publisher's Note

All claims expressed in this article are solely those of the authors and do not necessarily represent those of their affiliated organizations, or those of the publisher, the editors and the reviewers. Any product that may be evaluated in this article, or claim that may be made by its manufacturer, is not guaranteed or endorsed by the publisher.

